# 1461. Risk Factor Evaluation and Performance Improvement for Surgical Site Infections in Patients Undergoing Abdominal Hysterectomy at a Large Academic Public Safety Net Hospital

**DOI:** 10.1093/ofid/ofad500.1298

**Published:** 2023-11-27

**Authors:** Anna Buford, Tyler D Anderson, Roman Jandarov, Joseph Schaffer, Jacqueline Wells, Marianne Bartlett, Latitia Houston, Calvin White, Laura Buford, Madhuri Sopirala

**Affiliations:** UT Southwestern, Dallas, Texas; UT Southwestern, Dallas, Texas; University of Cincinnati College of Medicine, Cincinnati, OH; UT Southwestern, Dallas, Texas; Parkland Health, Dallas, Texas; Parkland Health, Dallas, Texas; Parkland Health, Dallas, Texas; Parkland Health, Dallas, Texas; Parkland Health, Dallas, Texas; UT Southwestern Medical Center and Parkland Health, Dallas, Texas

## Abstract

**Background:**

Incidence of deep and organ space surgical site infections (SSI) after abdominal hysterectomy ranges from 1.1% - 5.7%, with higher rates for women without private insurance. Parkland Hospital is one of the largest safety net hospitals in the United States, serving mostly uninsured and Medicaid-enrolled patients. In 2021, our deep and organ space SSI rate for abdominal hysterectomies was 1.39%, with a standardized infection ratio (SIR) of 1.456. We studied SSI risk factors and report performance improvement (PI) initiative that decreased SSI incidence.

**Methods:**

This retrospective case-control study randomly matched 37 patients with deep and organ space abdominal hysterectomy SSI from 2019 to 2021 with controls undergoing the same procedure during the same calendar month. We evaluated 18 variables as potential risk factors (Tables 1&2). We conducted a multidisciplinary PI intervention focusing on a modifiable risk factor identified during our investigation to reduce SSI post-hysterectomy. Evaluation showed vaginal and enteric organisms causing SSI leading to observations indicating improper vaginal preparation even though it was being performed.

**Table 1.**
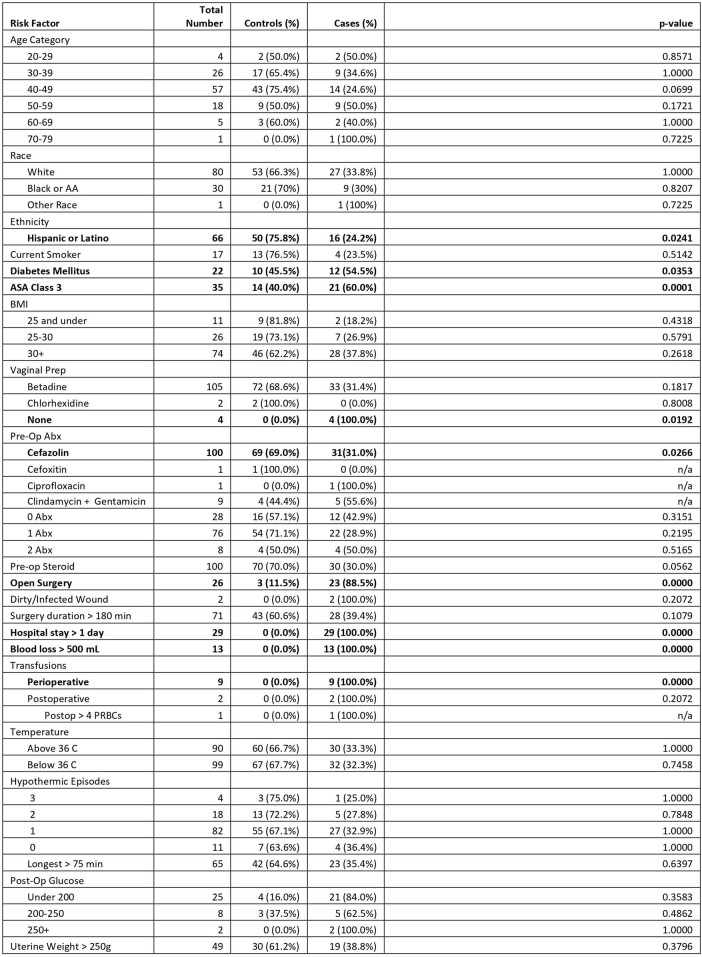
Risk factors for SSI after Abdominal Hysterectomy (Univariate Analysis)

**Results:**

Univariate analysis found one variable to be associated with lower risk and eight variables to be associated with higher risk (Table 1). Diabetes (OR, 3.89; 95% CI,1.25-12.08; p=0.0188) was found to be a risk factor in multivariate analysis while being Hispanic seems to be protective (OR, 0.31; 95% CI,0.12-0.80; p=0.0160). After the intervention of educating nursing staff on the right way of doing vaginal preparation, and weekly OR observations, our deep and organ space SIR in 2022 decreased to 0.63. Vaginal preparation was found to be a risk factor in our univariate analysis, but the coefficient was not estimable in multivariate analysis.

**Table 2.**
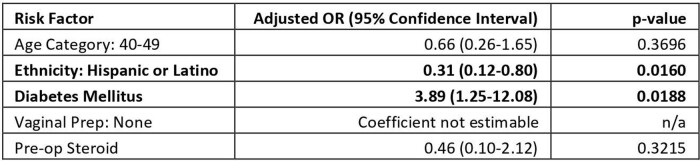
Independent Risk Factors for SSI After Abdominal Hysterectomy (Multivariate Logistic Regression)

**Conclusion:**

Multidisciplinary intervention significantly decreased deep and organ space SSI rates. Ensuring standardized pre-operative vaginal preparation also enhanced SSI risk reduction. We suspect that our intervention improved SSI rates by improving the accuracy of vaginal preparation. Performance of vaginal preparation was a risk factor in univariate analysis but was not significant on multivariate analysis.

**Disclosures:**

**All Authors**: No reported disclosures

